# Update on deep brain stimulation in Parkinson’s disease

**DOI:** 10.1186/s40035-015-0034-0

**Published:** 2015-06-27

**Authors:** Daniel Martinez-Ramirez, Wei Hu, Alberto R. Bona, Michael S. Okun, Aparna Wagle Shukla

**Affiliations:** Department of Neurology, University of Florida, College of Medicine, Center for Movement Disorders and Neurorestoration, 3450 Hull Road, Gainesville, FL 32607 USA; Department of Neurosurgery, Psychiatry, and History, University of Florida, College of Medicine, Center for Movement Disorders and Neurorestoration, Gainesville, FL 32610 USA

**Keywords:** Movement disorders, Subthalamic nucleus, Globus pallidus, Neural network, Brain modulation, Closed-loop

## Abstract

Deep brain stimulation (DBS) is considered a safe and well tolerated surgical procedure to alleviate Parkinson’s disease (PD) and other movement disorders symptoms along with some psychiatric conditions. Over the last few decades DBS has been shown to provide remarkable therapeutic effect on carefully selected patients. Although its precise mechanism of action is still unknown, DBS improves motor functions and therefore quality of life. To date, two main targets have emerged in PD patients: the globus pallidus pars interna and the subthalamic nucleus. Two other targets, the ventralis intermedius and zona incerta have also been selectively used, especially in tremor-dominant PD patients. The main indications for PD DBS have traditionally been motor fluctuations, debilitating medication induced dyskinesias, unpredictable “off time” state, and medication refractory tremor. Medication refractory tremor and intolerable dyskinesia are potential palliative indications. Besides aforementioned targets, the brainstem pedunculopontine nucleus (PPN) is under investigation for the treatment of ON-state freezing of gait and postural instability. In this article, we will review the most recent literature on DBS therapy for PD, including cutting-edge advances and data supporting the role of DBS in advanced neural-network modulation.

## Introduction

Parkinson’s disease (PD) is a chronic progressive neurodegenerative disorder affecting multiple brain circuits leading to motor symptoms such as bradykinesia, rigidity, resting tremor, and loss of postural reflexes [1]. PD also has non-motor manifestations such as neuropsychiatric symptoms, cognitive abnormalities, autonomic disorders, sleep and gastrointestinal problems [2]. The crude incidence rate of PD varies from 1.5 to 19 per 100,000 population per year [3]. The crude prevalence rates have shown steady age-related increase, shifting from 41 per 100,000 in the 40–49 age group to 1903 per 100,000 in people older than age 80 [4]. PD incidence and prevalence are expected to grow over next decades because of increasing efforts at extending the lifespan. Among the chronic disorders, PD is also considered one of the most difficult and challenging syndromes, and besides affecting a patient’s quality of life [5], it substantially increases caregiver burden [5].

Since levodopa was introduced by George Cotzias in 1967 [6], remarkable pharmacotherapy improvements have been made and new drugs and therapeutic strategies have been brought to the marketplace. However with progression of disease and development of drug-induced motor fluctuations and dyskinesias, surgical therapies have assumed an important role in treatment of this selected group of patients [7]. PD approved brain surgical treatments include deep brain stimulation (DBS) and stereotactic ablation; two procedures which aim to modulate abnormal neuronal activity within a circuit, and alleviate symptoms [8]. Our review focuses on DBS, highlighting the important aspects of this therapy, and discussing the historical aspect of brain focused electrical stimulation, We also discuss the biology and mechanisms of action related to DBS, the current state of cutting-edge methods to deliver electricity to the brain and important future directions.

## Review

### History of DBS

Ancient Egyptians used a fish capable of generating powerful current discharge to treat some types of pain [9]. Roman physician Scribonius Largus was credited with the first medical use of electricity describing in his text a number of remedies using an electric torpedo fish [10]. Many experts attribute much of the underlying basis supporting neurostimulation to Michael Faraday, a British scientist who discovered that an electrical current can produce a magnetic field [11]. In the 20th century, different types of stereotactic procedures between the 20’s and 60’s were developed around the world and ablative surgery was predominantly used to treat movement disorders symptoms and to alleviate some psychiatric symptoms. After the introduction of levodopa for PD in the mid 1960’s, this new drug temporarily ended the era of ablative surgery. However, the emergence of levodopa related complications and recognition of symptoms refractory to levodopa, forced the development of better treatments in spite of the new dopamine based approach. Lesional therapy studies in non-human primate MPTP models of PD also has led the field to a clearer understanding on the pathophysiology of PD and a better rationale for surgical intervention. In 1987 the French neurosurgeon Alim Benabid introduced the “chronic” high-frequency DBS approach and opened the door to a new era of human treatment for PD [12]. Over the past two decades a renaissance of neurosurgical treatments has emerged for both neurological and neuropsychiatric disorders.

### Theoretical biology and mechanisms of action

DBS is considered a well-established therapy for PD [13], even if it’s still unclear as to all of the biology and the actual mechanisms of action that underpin its benefits. Several theories have been proposed, implying that more than one mechanism is likely responsible for the therapeutic benefit.

Benabid’s group hypothesized that electrical stimulation induced an inhibition of basal ganglia output structures, decreasing basic firing of neurons and suppressing spontaneous neuronal activity [14, 15]. A reduction in activity in brain tissue surrounding the electrode, the possibility of depolarization blockade through K+ mediated effects [16], Na + channels inactivation [17], pre-synaptic depression of excitatory afferents [18], and hyperpolarization of neuronal bodies and dendrites [19] have all been reported. Glutamate reduction with concomitant increases of inhibitory neurotransmitters such as GABA [20] and adenosine [21] may also play a role in the biology and mechanisms of action of DBS.

Several studies have suggested an excitatory output from the stimulated neuronal target as another potential mechanism [22]. This idea has been supported by an observed increase in excitatory neurotransmitters [23]. Previous studies have shown that electrical stimulation induced an increase in blood flow in the globus pallidus internus (GPi) during subthalamic nucleus (STN) stimulation and also that cortical blood flow changes during thalamic stimulation. These studies are consistent with the activation of output hypotheses, and are further supported by PET and fMRI studies [24, 25], which have revealed correlations with Parkinson’s disease motor symptom improvement [26]. Furthermore advanced computational studies showed a possible simultaneous cell body inhibition mechanism with an axonal excitation [27]. Astrocytes (e.g. calcium release) are thought to play a role in this orchestra of changes including inhibiting cells, exciting fibers, changing cerebral blood flow, releasing excitatory neurotransmitters, and also stimulating neurogenesis [28]. The biological changes however, have not all been linked to mechanism of action. This simultaneous excitatory and inhibitory theory of DBS mechanism or DBS induced de-coupling, suggests that modifying the network activity and some of the overall positive benefits may underpin this therapy [29].

Beside these theories, it has been recently proposed that electrical stimulation may generate an “information lesion” by disrupting pathologic oscillatory patterns, preventing or disrupting the pathological basal ganglia activity from being transmitted [30]. This effect could be achieved by replacing irregular bursting cells, with regular high-frequency firing, and/or by promotion of “prokinetic” frequencies and abolition of pathological beta-band frequencies [31]. A recent study found that the electrical stimulation of the STN in PD patients using therapeutic parameters and a DBS electrode did not result in a profound or long-lasting inhibition of surrounding neurons, partially normalizing or “jamming” the pathological signal in the basal ganglia-thalamocortical network [32–34].

Recently, researchers have been looking at motor cortex activity when attempting to regulate motor function with DBS [35]. An emerging hypothesis is that the benefit of DBS is derived from direct modulation of primary motor cortex (M1) as observed in non-human primate, where STN DBS influenced both motor performance and M1 neuronal activity systematically according to stimulus intensity [36]. A recent study by Starr and colleagues showed that decoupling of the neuronal network and beta oscillations between STN and M1 was correlated to motor improvement [37].

Overall, it is currently believed that the application of electricity to the brain has definite inhibitory components, especially on neuronal cell bodies close to the origin of the current however there are important excitatory mechanisms at work as well. Although it remains unknown what the mechanisms underlying the therapeutic effects of DBS are, it is likely that DBS results in inhibition of the cell bodies close to the electrical field, and excitation of the axons. DBS could induce neurochemical changes that may be induced by stimulation of astrocytes and propagation of calcium waves resulting in release of chemicals such as adenosine and glutamate [28]. Important neurovascular and neurogenic changes may be induced by electrical stimulation of the brain as well [24, 25]. It is now clear that stimulation of small nodes can result in a large network-wide changes that may profoundly improve Parkinson’s disease and other movement disorders [38]. Additionally, important changes in oscillatory behavior (i.e. beta and theta bands) have emerged as clues to the underlying pathophysiology [39, 40].

### Current and new DBS targets

Chronic degeneration of dopamine neurons in the SNc and consequent striatal dopamine deficiency seems to lead to a cascade of functional changes, which underpin many clinical features of PD [41]. Studies published in the early 1990s using MPTP primate models of parkinsonism support the hypothesis that abnormally increased activity in STN leads to abnormal GPi activity [42]. This seems to lead to excessive inhibition of the thalamocortical [43] and brainstem motor projections [44].

Randomized, controlled, clinical trials have shown that DBS therapy can be superior to the best medical therapy for improving motor function and quality of life when carefully applying the therapy to a select group of PD patients [45–48]. The most important factor for successful outcomes is appropriate patient selection and in the last years, the concept of “tailoring” DBS according to a patient’s needs has been increasingly recognized [49]. Delivering electricity into the basal ganglia, specifically the STN or the GPi has replaced in most cases the initially studied nucleus, the ventral intermediate nucleus (VIM) of the thalamus. VIM DBS remains a valid option in select PD patients who have tremor as the main and disabling feature [7]. A multi-target strategy of stimulation could be a promising approach in the future, and several groups have used “rescue leads” when inadequate symptom relief has been achieved [50, 51]. Impulse control disorders and dopamine dysregulation syndrome have to be considered carefully because these conditions have been reported to be affected by DBS. Some studies have reported them as complications of DBS [52, 53], while others have observed an improvement after surgery, probably related to a reduction of dopamine agonist dosage [54]. Patient’s phenotype also has to be considered since a “brittle levodopa response” can be seen particularly in female candidates with low body weight. These patients have been reported to have a good response to DBS [55]. New targets are currently being investigated to treat symptoms that are less responsive to standard DBS targets such as the centromedian thalamus, zona incerta ZI, and the pedunculopontine nucleus [56]. The pedunculopontine nucleus (PPN) [57], and more recently the substantia nigra [58] have been used in an effort to improve gait and balance, however more carefully controlled studies will be needed to assess the potential benefits and risks as well as the appropriate selection criteria. Stimulation of the motor cortex has also been proposed as an alternative strategy, but the results have been inconsistent and with current methods in general have been disappointing [59]. Finally cerebellar outflow pathways have been suggested as possible targets as well however …….. [60].

### DBS controversies

#### Best target

Four randomized controlled trials have compared targeted therapies to STN and GPI and all have provided similar clinical results. In the extended study by Anderson and colleagues [61], no significant differences were found regarding the UPDRS motor scores (30 SD = 17 vs. 27 SD = 11; *p* = 0.4) when comparing GPi vs STN DBS at 12-months post DBS surgery. However, a tendency was observed for more levodopa reduction (38 % vs. 3 %), although greater cognitive and behavioral issues were seen in the STN group. Additionally, dyskinesia improved more in the GPi group (89 % vs. 62 %).

The COMPARE trial revealed no significant difference in motor outcomes between the two targets (*p* = 0.08, and *p* = 0.16, respectively) [62]. Furthermore, no UPDRS motor subscale difference was found between the two groups (STN 29.9 % vs. GPi 26.6 %; *p* = 0.64). However, word finding and letter verbal fluency worsening was observed (*p* < 0.03) and an increase in anger was observed particularly in the STN target group. In the follow-up studies on this cohort, patients receiving GPi DBS reported greater improvements in their quality of life as compared to the STN group (38 % vs. 14 %; *p* = 0.03) [63]. In addition, no differences in weight changes between targets were observed (STN 4.29 SD = 6.79 vs. GPi 5.38 SD = 10.32 lb.; *p* = 0.68) [64].

In the CSP 468 Study Group article (VA Multi-center VA study) [65], changes in UPDRS-III motor score did not differ significantly between study groups (−11.8 SD = 2.3 vs. 10.7 SD = 2.2; *p* = 0.50). Secondary outcomes did however reveal significant changes. Patients with subthalamic stimulation required lower doses of dopaminergic agents (165.4 SD = 143.7 mg difference; *p* = 0.02), processing speed declined more after subthalamic stimulation (2.5 SD = 2.2 difference; *p* = 0.03), and depression worsened after subthalamic stimulation, but improved with pallidal stimulation (*p* = 0.02). Serious adverse events were more common in patients undergoing subthalamic stimulation. Therefore, the authors concluded that non-motor factors should be included in target selection for DBS. In their 36-month follow-up study [66], motor function was better than baseline and there were similar improvements between targets (GPi 41.1 to 27.1 vs. STN 42.5 to 29.7; *p* = 0.59), however dementia scores declined faster for STN than GPi patients (*p* = 0.01). This study provides evidence that improvement in motor symptoms could possibly remain stable for several years post-DBS in a subset of patients.

In the most recent paper, the NSTAPS study, no significant differences were observed at 12 months in primary outcomes between STN and GPi DBS: disability and number of patients with a composite score of cognitive, mood, and behavioral effects [67]. Secondary outcomes that showed greater improvements in the off-drug phase favoring the STN group in the change in UPDRS motor scores (20.3 SD = 16.3 vs. 11.4 SD = 16.1; *p* = 0.03), change in ALDS scores (20.3 SD = 27.11 vs. 11.8 SD = 18.9; *p* = 0.04), and levodopa equivalent dose reduction (546 SD = 561 vs. 208 SD = 521; *p* = 0.01). The authors concluded that STN could be the preferred target for DBS in some patients with advanced PD.

Collectively and based on these results, our group recommends that the DBS target should be tailored on individual patient’s needs [68]. Cognition, behavior and dyskinesias for example should all be factors that are weighed in a decision for a best target. These discussions are best accomplished by the use of a multidisciplinary screening team.

### Earlier DBS

Earlier PD DBS intervention has been recently addressed. The EARLYSTIM Study [69] was a 2-year trial on PD patients with 7.3 (SD = 3.1) years of disease duration and with very early motor complications (dyskinesia 1.4 SD = 0.8 and motor fluctuations 1.6 SD = 0.8 mean years of duration) who underwent bilateral STN DBS plus best medical therapy, or alternatively a second group was randomized to best medical therapy. For the primary outcome of the change in PDQ-39 QoL, the mean score in the DBS group improved 7.8 points (26 %) vs. the medication group, which worsened by 0.2 points (1 %). These results suggested that STN DBS was superior to medical therapy in patients with PD and early motor complications. The study however enrolled only young patients with an average age of 52.9 (SD = 6.6), and therefore it was unclear how these findings would generalize to older populations especially those in their 60’s and 70’s. Vanderbilt University is currently conducting a prospective, randomized, single-blind clinical trial of optimal drug therapy (ODT) as compared to medication plus DBS (ODT + DBS) in subjects with early stage idiopathic PD without motor fluctuations or dementia [70]. The safety study was recently published, providing evidence that DBS is well tolerated in early PD [71].

### Utility of intraoperative microelectrode recording (MER)

Some groups have determined the final DBS lead location by relying on image targeting only, but the majority now use intraoperative microelectrode recording in order to define an optimal lead location. Physiology (microelectrode or semimicroelectode recordings, microstimulation) can be used to map and to localize a DBS target, and behavioral responses can be recorded as well. Factors in favor of using these targeting methods to refine electrode location include: 1- imaging inaccuracy or distortion, 2- inaccuracy of frame or frameless-guided navigation, and/or 3- brain shift due to cerebrospinal fluid loss and brain atrophy. The number of microelectrodes and the technique (target verification vs. mapping) is widely variable between centers. Microelectrodes generally have impedances greater than 0.5 MΩ and are capable of isolating single neural unit activity, and thus are useful in defining individual neurons within thalamic and basal ganglia nuclei. It is possible to deliver stimulation through microelectrodes. Semi-microelectrodes have impedances of approximately 100 kΩ and can provide information on group neural activity and local field potentials; they can also be used for stimulation. Macroelectrodes are low-impedance electrodes that can be used for recording field potentials or for local tissue impedance, but are used almost exclusively for stimulation and clinical responses. Response of the recorded signal to passive movements of the face or limb can confirm the location of the motor sub-territory of an intended brain target (STN, GPi or thalamus). Additionally a physiological response to a light directed to the eyes can aid in determining the location of the optic tract in GPi internus targeted cases. Stimulation can also be used to reveal the proximity of the lead or microelectrode to the motor fibers (i.e. internal capsule). Whilst MER is thought to slightly increase the risk of bleeding [72] and therefore many experts have been more judicious in the number of passes utilized, a recent study reported no bleeding complications on 590 MER tracks performed, suggesting that a carefule pre-surgical planning and a sequential MER approach could prevent this complication [73]. There are now several MRI and imaging based technologies in trial that do not use MER.

### DBS complications

Deep brain stimulation surgery is well tolerated and its complications rate is very acceptable from the “elective neurosurgery” standpoint. Complications can be categorized in surgery-related and hardware-related. First category consists of intracranial hemorrhage (with a mean incidence of 1.9 % in multicentric studies) [45, 65, 74, 75], seizures (mean incidence of 1.3 % in multicentric studies) [45, 74, 75], intraoperative bradycardia and hypotension, deep venous thrombosis and pulmonary embolism (0.7 % incidence in a multicentric study) [75], and pneumonia due to aspiration (incidence vary between 1 and 4 % in multicentric studies) [45, 65]. Second category consists of infection (incidence vary from 0 to 15.2 % among different groups) [76–80], skin erosion (incidence vary from 1 to 16 % among different groups) [81–83], DBS-lead migration and fracture (5 % incidence) [84].

### Emerging DBS technology

DBS as a surgical treatment for PD and other movement disorders has been historically delivered in an open-loop fashion, meaning in a pre-programmed chronic and continuous stimulation pattern. Newer or “smarter” delivery devices have been designed and have emerged to improve clinical efficacy, to decrease stimulation-induced side effects, and to prolong battery life.

Scheduled DBS delivery systems can be personalized to the frequency and duration of the manifestation or symptom of a particular disorder, such as in epilepsy [85]. A recent proof-of-concept study used scheduled DBS technology for Tourette syndrome (TS) tailoring stimulation pulse trains with long time intervals introduced between on and off stimulation revealing significant improvements in the clinical scores (mean change −17.8 SD = 9.4) of 5 patients after 6 months follow-up [86]. Although promising for movement disorders presenting in episodic pattern such as TS, further and larger studies will be necessary to confirm these results across other diseases and symptoms. It is unknown if the scheduled DBS system can provide better clinical outcomes than the chronic and continuous DBS system in the PD population. Additionally changing the shape of the pulse and using biphasic pulses (author observations) may be another avenue to improve outcomes.

Impedance is the amount of voltage required to deliver electricity divided by the resistance (Ohm’s law), and plays a critical role in how stimulation is delivered to the brain. Constant-current DBS systems are devices that hold impedance steady [87]. In the first study assessing the safety and efficacy of constant-current DBS, 136 PD patients underwent bilateral STN implantation and were randomly assigned to receive immediate vs. delayed stimulation [88]. Both groups revealed an increase in the duration of good quality “on” time after 3 months, significantly greater in the stimulation group (difference 2.51 h, *p* = 0.003) and UPDRS part-III scores improved by 39 % from the baseline in immediate stimulation group. A recent single-center study comparing safety and clinical impact of constant-current vs. constant-voltage STN DBS in PD patients, revealed no significant differences in motor scores between the electricity delivery methods after 2 years of surgery [89]. Although these were small studies, constant-current devices seem to be a promising method to delivery electricity to a changing brain. There is little information available on direct comparisons between voltage and constant current devices, and there may also be advantages of constant current in high current density situations (dystonia).

The concept of current steering refers to the use of multiple stimulation sources to direct current flow through targeted regions of brain tissue. There are several new systems where segmented electrodes will be able to shape the electrical field to activate targeted neural pathways without activating unwanted side effects [90, 91]. A recent single-center, performance and safety study where 8 PD patients underwent bilateral STN implantation, showed that stimulation with a novel 32-contact electrode was safe, capable of reproducing effects equivalent to standard electrodes, and could selectively and positively influence the thresholds for programming [92]. Whilst this study suggested that current steering devices could improve DBS effectiveness and limit side effects, more studies are needed.

Responsive or adaptive DBS systems have focused on interpreting brain electrical or neurochemical activity and use it as feedback to control delivery of therapeutic electrical stimulation [93, 94]. This cutting-edge technology is designed to “close the loop” by adjusting stimulation parameters according to the neural feedback recorded either coming from deep brain structures or from cortical feedback [94]. Recent proof-of-principle studies have shown that by personalizing and optimizing stimulation parameters in real time, efficacy and efficiency of continuous DBS could potentially be improved [95–98]. The clinical trial BrainRadio (NCT01990313) is currently enrolling patients and is using the Model 37604 Activa PC + S (Medtronic, Inc.) which is a multiprogrammable device that can deliver therapeutic electrical stimulation and record bioelectric signals from leads implanted in the brain. Our group at the University of Florida is currently recruiting patients to explore DBS in two specific brain regions (GPi + PPN) for freezing of gait in PD (NCT02318927). These studies will help us better understand the pathophysiology of the disease and hopefully it will improve clinical outcomes.

To date, DBS has been shown to be an effective therapy for selected patients with PD. There have been rapid advances in neuroengineering, and new “smart” DBS stimulation delivery systems are in development to improve the effectiveness and efficacy of this therapy.
